# The accuracy of laser fluorescence (DIAGNOdent) in assessing caries lesion activity on root surfaces, around crown margins, and in furcations in older adults

**DOI:** 10.1038/s41405-021-00069-2

**Published:** 2021-03-23

**Authors:** Chelsea Mitchell, Hiba Zaku, Peter Milgrom, Lloyd Mancl, David B. Prince

**Affiliations:** 1 Roseman University of Health Sciences, College of Dental Medicine, South Jordan, UT USA; 2 Department of Oral Health Sciences, University of Washington, Seattle, WA USA

**Keywords:** Gerodontics, Special care dentistry

## Abstract

The authors conducted a case series to assess accuracy of DIAGNOdent (DD) in assessment of activity of dental caries lesions in root surfaces and in furcations and at crown margins. The study was a prospective, single center case series. The patients were 123 adults (age ≥ 55 years). To be included, a patient needed to have at least one active root caries lesion. The study was conducted at the Roseman College of Dental Medicine in South Jordan, Utah, USA and at area nursing homes. Lesions were rinsed and dried with air, and DD readings were obtained. Lesions were then isolated and 38% silver diamine fluoride was applied repeatedly for two minutes with a microbrush. DD readings and treatments were repeated every six months. Mean DD values were significantly different between active (unarrested) and inactive (arrested) caries for all comparisons, *p*-value < 0.0001. The optimal cut-off values for DD were between 20 and 35 except optimal cut-offs were higher for furcation and crown margin surfaces, particularly in the posterior (optimal cut-offs 40–45). This study demonstrates DD is a potentially valuable tool for assessing lesion activity in root surfaces, at restoration margins, and in furcations.

## Introduction

The DIAGNOdent (DD) is a commercially available laser fluorescence device that includes a sensor that measures changes in back-scattered fluorescence to detect caries lesions.^1,2^ The fluorophores have been identified as bacterial porphyrins. A systematic review that included only studies where the caries diagnosis was validated with histological findings concluded that the DD was more sensitive than traditional visual–tactile methods; however, the studies were primarily conducted in vitro with limited generalizability to actual practice. The authors found increased likelihood of false-positive diagnoses compared with that of visual–tactile methods, and lesions were not followed over time as would happen in actual patient care.^3^

Only one study has examined the accuracy of DD to detect root surface lesions.^4^ In this study of 717 older adults, higher DD scores were associated with clinically active lesions. The authors found a cut-off between 5 and 10 on a scale from 0 through 99 resulted in the best combination of sensitivity and specificity. A major limitation of this work is that the results were not adjusted for clustering as individuals contributed more than one tooth surface to the analysis, which may have led to misleading results.

### Aims

The objective of this study was to assess the accuracy of DD in the assessment of activity in dental caries lesions in root surfaces and in furcations and at the margins of crowns. The study adhered to the guidelines in the Declaration of Helsinki and participants gave their written, informed consent. The Roseman University of Health Sciences, College of Dental Medicine Institutional Review Board approved the study (1122117-4). The study was part of a larger study assessing the effectiveness of silver diamine fluoride (SDF) treatment over time. The study registration number is NCT04370080.

### Design

The study is a prospective, single-center case series. The cases were consecutive.

### Setting

The study was conducted at the dental clinic of the Roseman College of Dental Medicine in South Jordan, Utah and in local nursing homes. The first patient was enrolled on December 6, 2016 and the last patient was enrolled on September 23, 2019. The last patient was evaluated at follow-up on March 13, 2020.

## Materials and methods

The patients were 122 older adults (age ≥ 55 years) who sought treatment at the clinic or were residents of local nursing homes. Students, faculty, and hygienists were trained and calibrated to the study protocol. The clinic patients were examined clinically by trained faculty members and dental students. The nursing home patients were examined by a licensed dentist and treated by two dental hygienists. To be included, a patient needed to have at least one active root caries lesion according to the criteria for caries activity published by Nyvad et al.^5^ with a DD score ≥20. Lesions were clinically confirmed and DD readings obtained for those lesions prior to intervention treatment. Lesions were both cavitated and non-cavitated. Patients were not excluded because of medical or psychological conditions in order to reduce selection bias and enhance the generalizability of the findings. Some individuals had more than one involved tooth and/or surface and each was included.

### Interventions

Lesions were treated with 38% SDF (Advantage Arrest, Elevate Oral Care, West Palm Beach, FL). The UCSF protocol for applying topical SDF was adapted for use with these patients.^6^ Lesions were flushed with water and then dried with compressed air, isolated with cotton rolls, and then 38% SDF was applied repeatedly for 2 min with a microbrush applicator. The SDF was stored under the manufacturer’s recommended conditions. SDF treatment was offered at no charge to the patient for the period of the study. Most enrolled lesions were deemed un-restorable and all patients chose the alternative treatment of SDF over extraction.

### Main outcome methods

The caries activity was assessed employing an adaptation of criteria developed by Nyvad et al., which have previously been shown to be reliable.^5^ To be scored as inactive, a root surface lesion had to be visible with the naked eye and feel hard with gentle pressure. There could be no pulpal involvement. The lesions were gently cleaned before the examination. The faculty members, students, and dental hygienists in the nursing homes were trained by the primary investigator (DBP) and co-authors (CM, HZ) using an illustrated slide presentation. Students were observed in the clinic under direct supervision to ensure consistent lesion detection. Lesions were re-examined by the same clinician.

Assessments were performed using a DD 2190 (KaVO, Biberach, Germany) initially and prior to SDF treatment at each subsequent appointment. Lesions were diagnosed using only the modified Nyvad criteria: DD was used to investigate the research question. Three machines were used throughout the study. The same DD machine was used for all readings for each patient. Machines 1 and 2 were used in the dental school clinic and machine 3 was used in nursing homes. Machines were calibrated according to the manufacturer’s instructions before each assessment. The distributions of exams across machines are given in Table 1. Before each reassessment and retreatment with SDF, the lesions were washed with water and then dried with compressed air. The teeth were isolated with cotton rolls. DD readings can range from 0 (completely inactive) through 99 (maximally active).

**Table 1:** Subject, tooth and tooth surface characteristics in a case series study of the accuracy of DD to assess dental caries activity in older adults.

	**Baseline**		**Follow-up**	
No. of subjects	122		97	
Age (year)				
Mean (SD)	76.0 (9.2)		77.5 (8.9)	
Median (IQR)	77.0 (69.3–83.0)		78.0 (71.0–84.0)	
Range	56–94		57–96	
	***n***	**%**	***n***	**%**
Sex				
F	64	52.5	51	52.6
M	58	47.5	46	47.4
DD machine				
1	44	36.1	34	35.1
2	56	45.9	42	43.3
3	22	18.0	21	21.6
No. of surfaces				
Mean (SD)	3.6 (3.5)		3.4 (3.6)	
Median (IQR)	2 (1–4)		2 (1–4)	
Range	1–16		1–16	
Total number	428		351	
	***n***	**%**	***n***	**%**
Tooth surface				
Distal	109	25.5	94	26.8
Facial	149	34.8	116	33.0
Lingual	80	18.7	69	19.7
Mesial	90	21.0	72	20.5
Tooth area				
Root surface	216	50.5	144	41.0
Furcation	13	3.0	12	3.4
Crown margin	199	46.5	195	45.6
Tooth position				
Anterior	157	36.7	138	39.3
Premolar	115	26.9	89	25.4
Molar^a^	156	36.4	124	35.3
Caries				
Active (unarrested)	428	100	57	16.2
Inactive (arrested)	0	0	294	83.8

^a^All surfaces, except 3, were from 1st and 2nd molars.

Clinical findings were entered into AxiUm (Exan, v. 7.02.01.58) by the examining student using the SOAP format and approved by clinical faculty members. Data were then abstracted from patient electronic records by one author (DBP) and entered into Excel (Microsoft Excel for MAC, version 16.37). Active (unarrested) and inactive (arrested, treated) lesions were contributed from both the baseline and follow-up periods in the evaluation of DD. The mean, standard deviation, median, and range were computed to describe the DD values for active and inactive caries. Mean DD values were compared between active and inactive caries using GEE linear regression, which accounted for the clustering of surfaces within a subject.^7^ Receiver operating characteristics (ROC) curve analysis was performed to estimate the area under curve (AUC) to assess the usefulness of DD for distinguishing between active and inactive caries lesions. AUC ROC curve results are considered excellent for AUC values between 0.9 and 1.0, good for AUC values between 0.8 and 0.9, fair for AUC values between 0.7 and 0.8, poor for AUC values between 0.6 and 0.7 and failed for AUC values between 0.5 and 0.6.^8^ Clustering of surfaces within a subject was accounted for using a nonparametric method for clustered ROC curve data,^9^ and tenfold cross-validation was used to estimate the ROC and AUC and the sensitivity and specificity for different DD cutoffs.^10^ Cross-validation was used to assess how the ROC curve analysis would generalize to an independent data set. ROC curve analysis was performed based on all surfaces and by DD machine, tooth surface, surface area, and tooth position. All statistical analysis was performed using R version 3.6.2.^11^

## Results

Ninety-seven of 122 (79.5%) participants were available for follow-up. The main reason for loss to follow-up was patients not returning to the clinic. The age and gender distribution of participants initially and at follow-up are given in Table 1. There was no significant difference in mean age or proportion of men and women between the two examinations. Lesion characteristics at baseline and follow-up are given in Table 1.

**Fig. 1:**
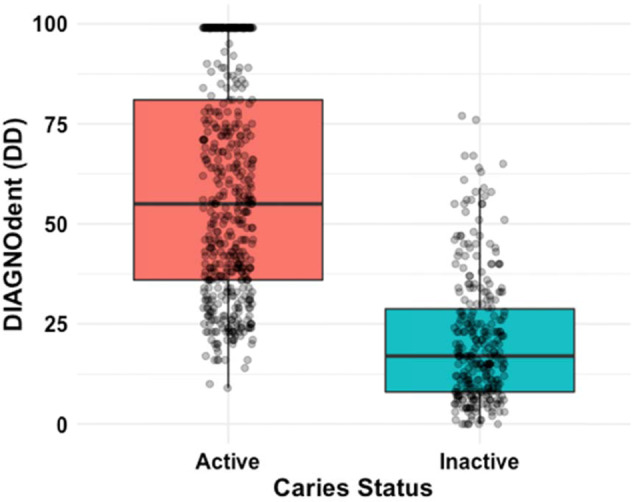
DIAGNOdent reading by dental caries status. DIAGNOdent (DD) scores by activity status of dental caries lesions at baseline and follow-up (*n* active = 428; inactive = 294).

DD scores for active (unarrested) at baseline and follow-up, and inactive (arrested) dental caries lesions overall, by DD machine, tooth surface, surface area, and tooth position are given in Table 2. Scores for machine 3 appear to be systematically lower than for the other machines. Mean scores for active surfaces (58.3 ± 27.2) were significantly higher than those for inactive surfaces (20.9 ± 16.1), even after adjusting for clustering of lesions within participants (Fig. 1, *p* < 0.0001).

**Table 2:** DD scores for active (unarrested) and inactive (arrested) dental caries lesions in an accuracy study of caries lesions in older adults.^a^

	Active (unarrested) caries at baseline				Active (unarrested) caries at follow-up				Inactive (arrested) caries			
Surface/DD machine	*n*	Mean DD ± SD	Median	Range	*n*	Mean DD ± SD	Median	Range	*n*	Mean DD ± SD	Median	Range
All surfaces	428	59.9 ± 26.7	56	20–99	57	46.4 ± 28.4	40	9–40	294	20.9 ± 16.1	17	0–17
DD machine												
1	145	60.2 ± 26.7	57	23–99	9	75.0 ± 30.0	90	29–99	85	24.6 ± 16.8	19	4–67
2	170	65.9 ± 26.0	66	20–99	15	50.8 ± 27.5	50	10–99	129	23.7 ± 16.0	21	0–77
3	113	50.6 ± 25.2	46	20–99	33	36.5 ± 22.6	28	9–99	80	12.5 ± 25.3	8	0–67
Tooth surface												
Distal or mesial	199	56.5 ± 26.4	52	20–99	24	35.9 ± 21.8	29	10–99	142	18.1 ± 15.9	13	0–77
Facial or lingual	216	60.0 ± 26.6	60	20–99	33	54.0 ± 30.4	46	9–99	152	23.5 ± 15.8	20	0–65
Surface area												
Root	216	60.0 ± 26.8	56	20–99	27	47.7 ± 30.3	41	16–99	168	15.8 ± 14.2	11	0–77
Furcation and crown margin	212	59.8 ± 26.6	56	20–99	30	45.2 ± 27.0	40	9–99	126	27.7 ± 16.0	24	3–76
Tooth position												
Posterior	271	60.8 ± 26.5	58	20–99	30	46.1 ± 26.8	40	9–99	183	24.5 ± 16.2	22	0–77
Anterior	157	58.4 ± 26.9	54	21–99	27	46.6 ± 30.5	38	10–99	111	15.0 ± 14.1	10	0–67

At follow-up, mean DD values were significantly different between active and inactive caries for all comparisons, *p* value <0.003. Mean DD values were not significantly different between active caries at baseline and follow-up for all comparisons (*p* value >0.05), except for DD machine 1 and distal or mesial tooth surfaces (*p* value < 0.01).

^a^Mean DD values were significantly different between active (baseline and follow-up combined) and inactive caries for all comparisons, *p* value < 0.0001.

**Fig. 2:**
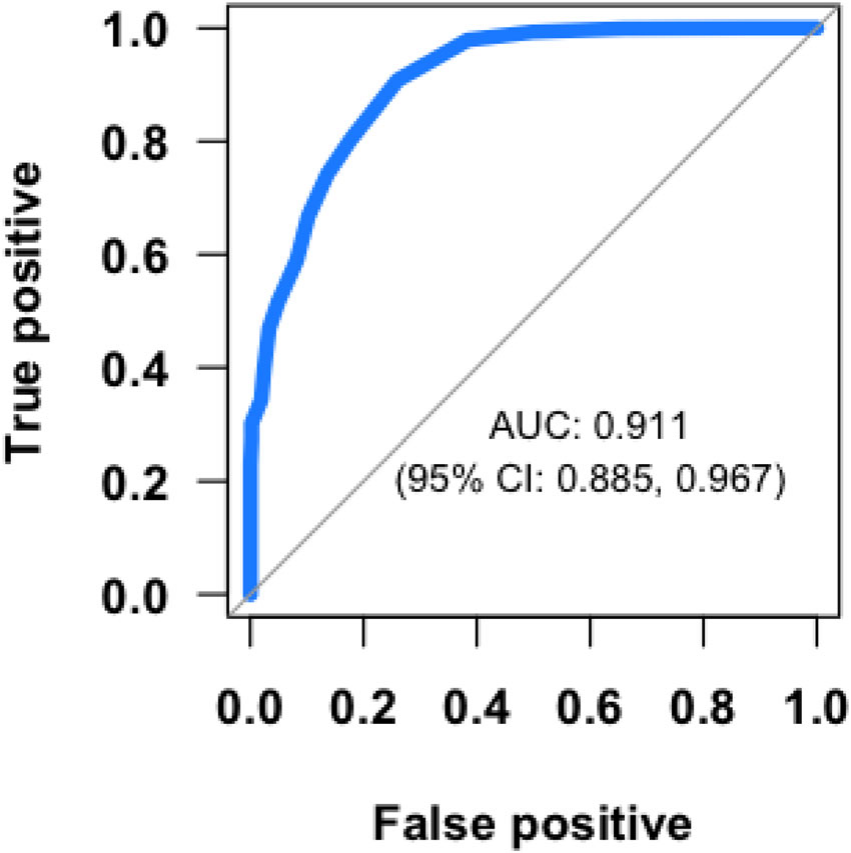
Relationship between true positive and false positive DIAGNOdent readings Receiver operating curve (ROC) and area under the curve (AUC) for DD based on baseline and follow-up for all surfaces (*n* active = 428; inactive = 294).

Table 3 gives the AUC, optimal DD cutoff, and sensitivity and specificity at the optimal cut-off for DD based on all surfaces, and by DD machine and tooth surface. Overall, for all surfaces, the AUC (0.911, 95% confidence interval 0.855–0.967) indicates outstanding discrimination between active and inactive lesions. For all surfaces, the optimal DD cutoff score is 25, taking into account the clustering of lesions within participants. The overall sensitivity (true positive) and specificity (true negative) were 90.8% and 74.0%, respectively, suggesting DD is effective in detecting active disease. Figure 2 gives the ROC curve and AUC for DD for all surfaces combined and illustrates the trade-off between sensitivity and specificity.

**Table 3:** Area under the curve (AUC) and sensitivity and specificity at the optimal cutoff for DD based on all surfaces and by DD machine and tooth surface.

Surface/Machine	AUC (95% CI)	Optimal DD cutoff	Sensitivity	Specificity
All surfaces	0.911 (0.855–0.967)	25	90.8%	74.0%
Machine				
1	0.926 (0.814–1.000)	30	93.8%	78.1%
2	0.921 (0.826–1.000)	30	89.9%	76.7%
3^a^	0.941 (0.872–1.000)	20	92.5%	77.9%
Tooth surface				
Distal or mesial	0.922 (0.836–1.000)	20	98.4%	75.8%
Facial or lingual	0.923 (0.842–1.000)	35	88.3%	83.6%
Surface area				
Root	0.954 (0.928–0.980)	25	89.1%	86.3%
Furcation and crown margin	0.829 (0.685–0.972)	45	67.9%	78.3%
Tooth position				
Posterior	0.899 (0.816–0.982)	40	72.4%	87.1%
Anterior	0.958 (0.910–1.000)	25	86.5%	88.9%

^a^Due the smaller sample sizes which produced training data sets with no variation in caries status (either all inactive or active caries) using tenfold cross-validation, fivefold cross-validation was used when the data were restricted to Machine 3.

## Discussion

The optimal cut-off values for DD overall and by machine, tooth surface, surface area, and tooth position were between 20 and 35 except that the optimal cut-offs were higher for furcation and crown margin surfaces, particularly in the posterior (optimal cut-offs 40–45). The accuracy of DD to detect active (unarrested) and inactive (arrested) dental caries lesions was excellent overall and good to excellent for all comparisons. The accuracy was higher for root surfaces when compared to furcations and crown margin surfaces, and also higher for anterior surfaces when compared to posterior surfaces (premolars and molars).

Previous studies have suggested that DD can be used to quantify the activity of tooth decay^12^ where more advanced lesions produce a distinctive fluorescence and higher DD readings.^1^ The DD has been used to detect the difference between inactive and active decay on root surfaces.^13^ It can also be used to assess remineralization after treatment with SDF.^14^ However, the findings of the present study differs from the previous study of root surfaces suggesting that the cut-offs recommended are too generous, and based on our study, the readings would lead to many false positives.

In this study, the lesions were already confirmed clinically, and the DD readings validated with the results of a visual–tactile examination. The staining of active lesions that occurs with SDF treatment may impact DD readings although the only data on this subject are from a very limited analysis of a tiny number of naturally discolored lesions in permanent teeth.^2,15^ These studies evaluating discoloration were largely done in vitro and were looking at the DD’s ability to assess occlusal decay in stained fissures.^15^ It is well understood that DD performs less reliably in fissures. Nevertheless, the same study validated the DD readings with histological findings of caries lesions. None of the teeth studied, however, had root surface lesions or recurrent caries at the margins of restorations.

### Limitations

The examiners in the study were carefully trained to well established criteria but the reliability of the clinical exams was not formally measured. Nevertheless, the high rate of arrest after SDF treatment reduces the measurement variability (unpublished data). The difference in the readings of the third machine are not explained, however, the results are still consistent with the hypothesis.

## Conclusions

This study advances our current knowledge demonstrating that DD is a potentially valuable tool for assessing lesion activity in root surfaces, at restoration margins, and in furcations. Additionally, cut-offs for DD determined in this study are more conservative than previously published and should mediate against false-positive findings.
